# Healthcare choices in Mumbai slums: A cross-sectional study

**DOI:** 10.12688/wellcomeopenres.13127.2

**Published:** 2018-07-06

**Authors:** Elina Naydenova, Arvind Raghu, Johanna Ernst, Sirazul A. Sahariah, Meera Gandhi, Georgina Murphy

**Affiliations:** 1 Institute of Biomedical Engineering, University of Oxford, Oxford, OX3 7DQ, UK; 2 Centre for the Study of Social Change, Mumbai, Maharashtra, 400051, India; 3 Centre for Tropical Medicine and Global Health, Nuffield Department of Medicine, University of Oxford, Oxford, OX3 7BN, UK

**Keywords:** healthcare access, India, slums, healthcare providers, maternal health, non-communicable disease, universal health coverage

## Abstract

**Background:** Informal urban settlements, known as slums, are the home for a large proportion of the world population. Healthcare in these environments is extremely complex, driven by poverty, environmental challenges, and poor access to formal health infrastructures. This study investigated healthcare challenges faced and choices made by slum dwellers in Mumbai, India.

**Methods** **:** Structured interviews with 549 slum dwellers from 13 slum areas in Mumbai, India, were conducted in order to obtain a population profile of health-related socio-economic and lifestyle factors, disease history and healthcare access. Statistical tools such as multinomial logistic regression were used to examine the association between such factors and health choices.

**Results** **:** Private providers (or a mixture of public and private) were seen to be preferred by the study population for most health conditions (62% - 90% health consultations), apart from pregnancy (43% health consultations). Community-based services were also preferred to more remote options. Stark differences in healthcare access were observed between well-known conditions, such as minor injuries, pulmonary conditions, and pregnancy and emerging challenges, such as hypertension and diabetes. A number of socio-economic and lifestyle factors were found to be associated with health-related decisions, including choice of provider and expenditure.

**Conclusions:** Better planning and coordination of health services, across public and private providers, is required to address mortality and morbidity in slum communities in India. This study provides insights into the complex landscape of diseases and health providers that slum dwellers navigate when accessing healthcare. Findings suggest that integrated services and public-private partnerships could help address demand for affordable community-based care and progress towards the target of universal health coverage.

## Introduction

By 2025, the world population is projected to reach 8.1 billion, with the majority of this growth taking place in developing countries ^1^. Nowhere else will this growth be more concentrated than in the already heavily populated informal settlements of cities known as slums. Slums are said to represent a fundamental transformation of the physical and social environment of cities and of human health ^2^. In India, over 100 million people are estimated to live in urban slums ^3^. The determinants of health in urban slums are extremely complex, a concentration of the detrimental effects of poverty and environmental challenges, as well as marginalisation from formal infrastructure and services ^4^. If India is to make progress towards achieving the Sustainable Development Goal 3 to ‘ensure healthy lives and promote well-being for all at all ages’, a focus on slum populations will be crucial ^5^.

In addition to persisting challenges of infectious diseases and maternal health, low-resourced settings are confronted with a growing burden of non-communicable diseases (NCDs). Low- and middle-income countries currently experience 82% of the global burden of premature NCD deaths, with 250 million slum dwellers estimated to suffer from NCDs ^6– 8^. The determinants of NCDs in urban slum populations are complex, with risk factors ranging from lifestyle, to infectious diseases, such as HIV and hepatitis, and to malnutrition and suboptimal foetal programming during pregnancy ^9, 10^. The latter is a particular challenge in Indian slums, where 40% of pregnant women are malnourished and the prevalence of gestational diabetes and gestational hypertensive disorders have been reported to be as high as 35% and 15%, respectively ^11– 13^. With appreciation for this interconnection, the UN declaration from 2011 advocates implementation of integrated programmes across NCDs and maternal and child health (MCH) ^14^. Yet, little is understood about how such integrated programmes could fit in the current fragmented landscape of health services in urban slums.

Agarwal has published a comprehensive overview of the healthcare landscape in Indian slums and the National Family Health Survey, conducted by the government of India, has reported on incidence statistics, particularly in the context of maternal and child health ^15, 16^. Beyond these, health statistics and access to care data about slum populations are sparse, with the vast inter- and intra- slum variability rarely captured in formal reports ^17– 20^. Identifying slums as spatial entities in data systems, including national censuses, is one of the main policy recommendations listed in a recent Lancet series on the health of people who live in slums ^20^. The lack of population-based data likely leads to underreporting of chronic conditions that remain undiagnosed, untreated, and hence unreported ^2^. Moreover, this sparsity of data results in poor understanding of the factors associated with healthcare decisions influencing choice of provider and healthcare expenditure. A few studies have suggested that slum communities access care from a mixture of public and private providers, with distance, household decision-making structure and perceived quality of care being important factors in their decision ^21– 24^. Association between socio-economic factors and availability, affordability and utilisation of health services have also been reported ^25– 27^. Without a better understanding of how slum populations navigate India’s complex healthcare environment, effective design of healthcare policy or implementation strategy to improve access to essential healthcare services and address current and emerging epidemics will be limited and challenging.

This study examined the disease burden, health awareness and services, and healthcare choices reported by residents across 13 slums in Mumbai, India. To provide insights into the heterogeneous nature of healthcare choices, the study focused on parents of young children who are likely to have recently encountered a number of health challenges including maternal health, common health conditions, and early-onset NCDs. These insights are of high relevance to integrated health programmes looking to align incentives behind traditionally verticalized services.

## Methods

### Study setting

The study took place between January and May 2015 in the Bandra, Khar, Santa Cruz, and Andheri areas of the city of Mumbai, India, in 13 slum areas covered by the health and social programs of the nongovernmental organization the Centre for the Study of Social Change (CSSC). The study population and health system context has previously been described ^13, 28^. Briefly, Mumbai is home to about 12.4 million people, with population density of about 19,652 people per km2 ^29^. Rapid migration from rural Maharashtra, and other states like Bihar and Uttar Pradesh puts a burden on Mumbai’s infrastructure and services. Approximately 41.8% of the population of Mumbai live in slums ^29^. Slum accommodation varies from simple shacks to permanent structures; they are all very close together and small, with a family typically living in one room approximately 8 × 10 ft. The CSSC works on grassroots social development in these communities, with a particular focus on primary healthcare services; since 1972, the organisation has built up an infrastructure of trained health workers that engage in both health delivery and research projects.

The study focused on parents of young children in order to provide direct comparison between people’s choices regarding a range of healthcare challenges. The demographic captured in this study is strongly indicative of future challenges facing urban slum populations as their current choices and risk factors will directly affect their and their children’s health. Moreover, integrated health solutions targeting early risk factors of NCDs, as highlighted in the Introduction, would target this demographic group.

### Data collection

Data collection was conducted by 22 community healthcare workers, with previous experience in community-focused research. Training for the interview process was conducted by CSSC senior research staff over a period of two days, followed by a series of mock interviews supervised by a senior member of the research team to ensure processes were followed correctly. Each interview lasted approximately 30 minutes and consisted of a series of standardised questions on: socio-economic background; disease history; risk factors, including nutritional patterns, smoking and alcohol consumption habits; access to health care for a range of conditions; and access to medication. The series of questions on NCD access were designed by appropriating the WHO recommended NCD STEPS survey ^30^. The Key Indicators of Urban Slums in India document, created by the Indian Ministry of Statistics and Programme Implementation, was consulted to ensure that all questions were contextually appropriate ^31^. The questionnaire was designed in English and translated in Hindi and Marathi by a local translator (translation was reviewed by senior research staff at CSSC) to accommodate language preferences across all participating communities.

The following procedure was followed in order to obtain a sample of the population: (1) the slums were divided into 13 areas, covering a population of approximately 65,000 people and 13,000 households; (2) each health worker was assigned an area to cover; (3) health workers were asked to approach people in their households at random, ensuring participants were not from the same or directly neighbouring households. Participation in the interview process was entirely voluntary and each participant was clearly informed of their right to withdraw at any point of the interview. Both male (152) and female (397) participants were interviewed; a larger group of women was interviewed since maternal health questions represented a substantial part of the interview.

The following were the participant eligibility criteria:

- be over 18 years of age;
- have lived in this area for at least 6 months;
- have had children within the last 5 years or be currently expecting a child;
- have not participated in recent large-scale healthcare studies and trials (to avoid bias in healthcare access perception)
- only one member of a given family or household was interviewed

Data were collected, digitised and stored by the CSSC. Anonymised data were shared with the research team at the University of Oxford for analysis.

### Statistical analysis

Data were analysed using statistical packages in Matlab 8.0 and Statistics Toolbox 8.1 (The MathWorks, Inc., Natick, Massachusetts, United States). Results are presented as median with interquartile range (IQR) for continuous variables and prevalence for categorical variables. Statistical significance tests were performed using the Mann-Whitney U test due to its nonparametric nature and applicability to ordinal data.

Multinomial logit modelling was used to examine the association of socio-demographic and lifestyle factors with choice of healthcare provider, where the use of public provider was taken as a baseline and the use of private or multiple providers represented the two other possible categories. Similarly, multiple linear regression was used to investigate the association of such factors with the cost of health consultations.

### Ethical statement

Ethical approval for the study was granted by the Oxford Tropical Research Ethics Committee (OxTREC Reference: 556-14). Written informed consent was obtained from all participants in the study at the time of interview.

## Results

### Population profile and lifestyle factors

Between January and May 2015, 557 people were invited to participate in the study; 549 agreed, representing a response rate of 99%. The socio-demographic characteristics of the study population are shown in Table 1. The study population was young, with a median age of 29 years (33 years for men and 28 years for women). The percentage of households earning less than 12,000 INR (USD 180) monthly was 76.6% and nearly 50% reported earned less than 9,000 INR (USD 135). Decisions regarding the household finances (including healthcare payments) were reported to be made primarily by the husband (62%) in the family or in some cases his parents (27%). The majority of women (83%) did not engage in any paid labour and instead performed unpaid work in the home; only 32% of men were in a permanent job, in contrast to 54% of men in temporary/casual labour. Some of the most common occupations amongst those employed were: driver, seller of goods, shop/stall owner, tailor and household assistant. Approximately half of the respondents reported to have completed Higher Secondary Education (52%), with no statistically significant differences found between men and women.

**Table 1.** Socio-demographic characteristics of population, reported separately for men and women.

	Men (N = 152)	Women (N = 397)
**Age** [years] ** *Median (IQR)*	33 (30 – 37)	28 (25 – 31)
**Residency in slum** [years] ** *Median (IQR)*	25 (7 – 33)	6 (3 – 11)
**People in household** *Median (IQR)*	5 (4 – 7)	5 (4 – 7)
**People in household in paid work** *Median (IQR)*	1 (1-2)	2 (1-2)
**Children** *Median (IQR)*	1 (1-2)	2 (1-3)
**Household monthly income** *n (% of all respondents)*		
< 6,000 INR (USD 90)	15 (10)	56 (14)
≥ 6,000 INR (USD 90) & < 9,000 INR (USD 135)	54 (36)	134 (34)
≥ 9,000 INR (USD 135) & < 12,000 INR (USD 180)	39 (26)	120 (30)
≥12,000 INR (USD 180)	43 (28)	85 (22)
**Types of occupation** ** *n (% of all respondents)*		
Unpaid work at home	1 (1)	329 (83)
Paid permanent job	48 (32)	11 (3)
Paid temporary/casual labour	80 (54)	42 (11)
Other	20 (13)	12 (3)
**Decision maker regarding household finances** ^a^ *n (% of all respondents)*		
Man/Husband	103 (68)	229 (58)
Woman/Wife	6 (4)	36 (9)
Maternal parents	0 (0)	12 (3)
Paternal parents	41 (27)	106 (27)
Other	2 (1)	12 (3)
**Completed education level** *n (% of all respondents)*		
None	1 (1)	16 (4)
Primary [1–5 years]	6 (4)	19 (5)
Secondary [6–8 years]	10 (7)	37 (9)
High School [9–10 years]	25 (17)	71 (18)
Higher Secondary [11–12 years]	84 (56)	202 (51)
University/Vocational Schooling	25 (17)	51 (13)

* p ≤ 0.01 ** p ≤ 0.001 for the difference between men and women.

Lifestyle characteristics of the population are reported in Table 2. Both alcohol consumption and tobacco use were seen to be more prevalent in men than women, 24% versus 0% (p<0.001) and 18% versus 5% (p<0.001), respectively. Self-reported levels of physical activity during work were higher in men (52%) than in women (32%) (p<0.001).

**Table 2.** Lifestyle characteristics and health awareness reported separately for men and women.

	Men (n = 146)	Women (n = 396)
**Fruit & vegetable** [portions per week] *Median (IQR)*	4 (3-5)	4 (3-5)
**Protein & carbohydrates** [portions per week] *Median (IQR)*		
Eggs	2 (1-3)	2 (2-3)
Meat	2 (2-3)	2 (1-3)
Beans	1 (1-2)	1 (1-2)
Lentils	7 (6-7)	7 (5-7)
**Alcohol consumption** ^a^ ** *n (%)*	36 (24)	0 (0)
**Tobacco use** ^b^ ** *n (%)*	27 (18)	21 (5)
**Physical activity during work** ^c^ ** *n (%)*	76 (52)	126 (32)
**Importance of clinical consultation during** **pregnancy** *n (%)*	149 (98)	397 (100)
**Disease awareness** *n (%)*		
Hypertension	106 (70)	259 (65)
Diabetes	116 (77)	283 (73)
**Awareness of sources of care** *n (%)* ^d^		
Pregnancy	397 (100)	
Hypertension	32 (53)	
Diabetes	12 (80)	

* p ≤ 0.01 ** p ≤ 0.001 for the difference between women and men. a: defined as non-abstainers; b: consumption of any type of tobacco; c: more than 10min of continuous physical activity; d: for pregnancy n=397; for hypertension n=61; for diabetes n=15.

### Maternal and NCD health awareness

Health awareness related to maternal health (MH), as well as hypertension and diabetes, was recorded ( Table 2). An overwhelming majority of both men (98%) and women (100%) reported that clinical consultation during pregnancy is important. A high proportion of participants were able to define hypertension (67%) and diabetes (74%). No statistically significant differences were found between the two sexes (hypertension: p=0.321, diabetes: p=0.283). Participants with higher levels of education were more likely to be aware of hypertension and diabetes as compared to those with lower levels of education (p<0.001 in both cases).

### Prevalence of disease

Most people reported to have experienced minor conditions (Category I: headache, minor burns and injuries) at some point in their life (71% of men and 75% of women). Experience of more complex conditions was lower: 15% for chronic conditions (Category II: pulmonary/cardiovascular disease symptoms, such as chest pain and breathing problems, and other chronic problems); and 9% for severe trauma (such as major injuries, bleeding and fractures). No statistically significant difference in disease history between men and women was observed (Category I: p=0.253, Category II: p=0.701, Category III: p=0.134). Experience of conditions from two categories was reported by 20% of respondents and experience of conditions from three categories by 5% of respondents. The self-reported current prevalence of hypertension was 10% amongst men and 12% amongst women (p = 0.572). The self-reported prevalence of diabetes was 2% amongst men and 4% amongst women (p=0.351). Participants reported higher prevalence of the two conditions amongst family members, 26% for hypertension and 20% for diabetes, with 10% of families affected by both conditions.

### Access to healthcare services

Access to a number of relevant health services was investigated. A higher proportion of women than men reported to have ever had their blood pressure and blood sugar measured - 48% versus 39% (p=0.062) and 25% versus 13% (p<0.001), respectively. Only 50% of participants with hypertension and 58% of those with diabetes had ever taken medication for their condition.

Within maternal health, access to services appeared to be good. Most women reported to be accessing some type of maternal healthcare during pregnancy. Although 94.5% of all women stated that visiting a clinic during pregnancy is important, 79.3% said it was too expensive and 75.8% said it was too far away. Consequently, 92.0% of women accessed maternal care during pregnancy but only 61.5% reported to visit a clinic, with the rest relying on providers in the community. Sixty three percent of women reported to have had their first consultation within the first trimester and 92% had a consultation at least once per month, after their first consultation. More than 90% of women reported to receive essential pregnancy services, including blood and urine tests, ultrasound scans, weight measurement and abdominal palpation. More than 85% of women reported to take pregnancy tablets such as folic acid, iron and vitamins during pregnancy.

### Choice of healthcare providers

**Figure 1.**
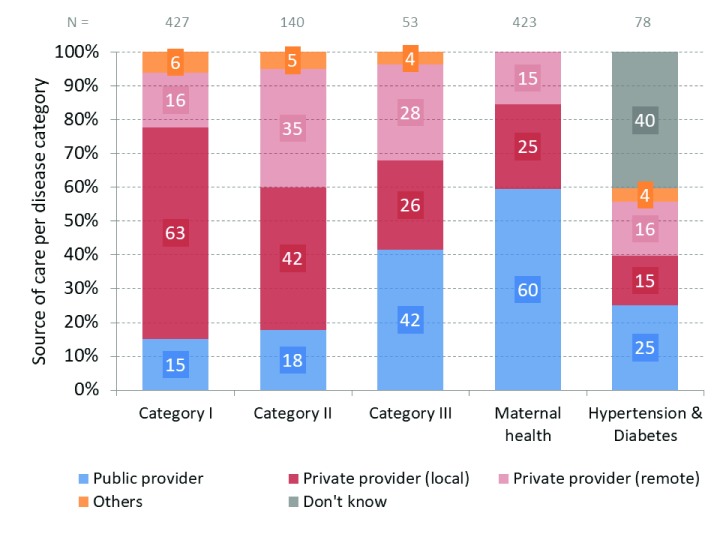
Types of providers per disease category. The population in each disease category includes the number of people affected by that condition. Participants were allowed to select multiple providers; therefore, for each disease category, n denotes the sum of responses for that disease category. Category I: Minor disease, e.g. headache, fever, minor burns, injuries. Category II: Pulmonary/Cardiovascular disease symptoms, e.g. chest pain & breathing problems as well as other chronic conditions that require multiple consultations. Category III: Severe trauma such as major injuries, bleeding, fractures.

The choice of healthcare providers was reported to vary depending on the disease, as illustrated in Figure 1. Results are reported on the basis of people who have been previously or are currently affected by a given condition. Participants could pick multiple providers per health condition, hence the percentages indicate the proportion that each type of provider represents of all answers pertaining to a category. Private providers were seen to dominate as the preferred source of care in all categories, apart from maternal health. Mild conditions and conditions requiring repeated visits were primarily dealt with through private providers locally. Treatment for more severe trauma was split between public (42%) and private (54%); surprisingly, local private providers were chosen by 26% of respondents even for this type of health problem. Public providers were seen to play an important role in maternal health (60%); 33% of women reported to access a mixture of both public and private services during pregnancy. Many people affected by hypertension and diabetes could not identify a provider that they can receive care from for their condition (40%). Contributions from providers of alternative care or pharmacies appeared to be minimal (≤6% across all conditions).

In cases where multiple visits were required to resolve the health issue, 56% of respondents reported to switch providers. For any given disease category or MH, a mixture of public and private providers was reported by less than 6% of respondents; yet, aggregated across disease categories and MH this resulted in 32% of people using a mixture of public and private providers.

Variation in provider choice was also observed regarding access to medication - 55% of women reported to procure pregnancy supplements through the public health system (public doctor, health post or community health worker) and the rest procured them from a private provider.

### Healthcare costs

Costs associated with health consultations for the health conditions investigated, across various provider types as reported by participants, are summarised in Table 3. The majority of governmental services were reported to be accessed for free or at very low cost (10 INR). The cost of care from a private provider locally was seen to be comparable across disease categories, with median values of 50 INR, 70 INR and 65 INR across Category I, II and III, respectively. By contrast, remote providers of private care, outside the community, were reported to charge a higher and more variable fee, depending on the type of condition, with median values of 60 INR, 200 INR, 500 INR for Categories I, II, III, respectively.

**Table 3.** Costs associated with each provider for a given disease category. The population in each disease category includes the number of people affected by that condition.

	Costs per consultation in INR - Median (IQR)			
	*Public provider*	*Private provider* *[local]*	*Private provider* *[remote]*	*Pharmacist*
Category I	10 (10-10)	50 (50-80)	60 (50-100)	10 (0-10)
Category II	10 (10-10)	70 (50-100)	200 (100-500)	50 (40-125)
Category III	10 (10-10)	65 (50-70)	500 (300-3500)	NA
	*Public providers*		*Private providers*	
Maternal Health	10 (0-10)		200 (100-460)	

Category I: Minor disease, e.g. headache, fever, minor burns, injuries. Category II: Pulmonary/Cardiovascular disease symptoms, e.g. chest pain & breathing problems as well as other chronic conditions that require multiple consultations. Category III: Severe trauma such as major injuries, bleeding, fractures.

Within maternal health, women paid for their pregnancy consultations in 73.5% of the cases. Payment in the public sector was minimal, median of 10 INR (IQR 10-10 INR), and payment in the private sector was considerably higher, median of 200 INR (IQR: 100-460 INR).

Given the small number of people suffering from hypertension and diabetes, the interview enquired about willingness to pay rather than history of payment as for the other conditions. More than 93% of all people interviewed reported being willing to pay to get tested for either of these conditions. Differences were observed in the amount people were willing to pay for hypertension testing, median of 20 INR (IQR: 10-30 INR), versus diabetes testing, median of 30 INR (IQR: 20-50 INR).

### Healthcare choices

Table 4 presents insights into participant characteristics and their associations with healthcare provider choice and cost. Women with higher income and those who reported higher fruit & vegetable consumption were more likely to choose private than public providers for maternal healthcare. For general conditions (i.e. disease Categories I, II and III combined), women from smaller households, women from households with higher income, women with more children, and men who reported consuming more portions of protein and carbohydrates were more likely to use mixed providers (public and private) compared with only using public providers.

**Table 4.** Association of socio-demographic and lifestyle determinants with healthcare choices.

	Provider (Odds Ratios)				MH provider (Odds Ratios)		Cost (coefficients)		MH cost (coefficients)
	Private		Mixed		Private	Mixed			
	Men	Women	Men	Women	Women	Women	Men	Women	Women
Age	1.02	1.01	1.01	1.01	1.05	1.09	0.04	-0.05	0.01
Residence	1.02	0.98	1.00	1.00	1.00	1.04	-0.03	-0.05 *	0.01
Occupation	2.82	0.80	0.96	0.59	0.64	1.24	0.03	0.00	0.00
Household size	0.71	0.87	0.82	0.68 *	0.90	1.22	0.01	0.02	0.02
Earning people	2.48	1.26	3.11	1.68	1.26	0.73	0.02	-0.01	-0.02
Household income	0.70	1.24	0.53	1.85 *	1.36 *	1.54	-0.04 *	0.00	-0.01
Education	2.42	1.50	4.48	1.31	1.26	0.85	0.05	0.00	0.01
Children	0.81	1.16	0.77	2.29 *	0.79	0.95	-0.01	0.01	-0.02 *
Fruit & veg	1.02	0.96	0.63	0.91	1.21 **	1.31 **	-0.01	-0.01	0.00
Protein & carbs	1.98	0.79	5.54 **	0.80	0.83	0.81	0.03	0.00	-0.03*
Alcohol	1.35	NA	2.40	NA	NA	NA	0.01	NA	NA
Tobacco	0.55	0.30	0.52	0.33	0.75	0.42	0.01	0.01	-0.02 **
Activity	0.59	0.79	0.77	0.45	1.12	1.07	0.01	0.00	-0.01

Provider and cost is used as an aggregate for choices pertaining to disease Categories I, II and III and maternal health has been presented separately. Multinomial logistic regression was used to derive odds ratios for a range of factors potentially associated with provider. Public provider was used as a baseline and private and mixed (private & public) as the two alternative options. Multiple linear regression with cost as the dependent variable was used to derive standardised coefficients associated with each factor. For both methods, only p-values that fall within two significance categories (*p≤0.05, **p≤0.01) are included for ease of interpretation.

The amount people chose to spend on individual consultations in the general disease category was seen to be associated with household income for men and residency in the community (in years) for women. Women with more children, those who reported consuming more protein and carbohydrates, and those who reported smoking tobacco, reported spending less on maternal health care.

## Discussion

Our study presents findings on healthcare challenges and choices in a number of slum communities across Mumbai. The study focused on parents of young children as a subsection of the population experiencing a range of health challenges, where healthcare choices are influenced by different socio-economic factors. A number of important differences with previous studies, focused on other demographic groups, were found, including differences in education levels and health awareness, as discussed below. This demographic is highly relevant for organisations designing integrated health programmes (e.g. NCD-MCH programmes) as well as forward-looking health policy development that might target this demographic as a household entry point for health interventions (more than 65% of participants reported to be responsible for financial decisions including healthcare of the household).

The population studied was found to have limited income (50% of households earned less than 9,000 INR (USD 135) per month, compared to a national minimum wage for unskilled labour of INR 9,568 ^32^); literacy in the community was high (the majority had high school education or above). Health awareness was found to be good and demand for healthcare service high, evidenced by: regular accessing of antenatal services, willingness to pay for diagnostic tests for diabetes and hypertension, as well as spending on private over public providers. Differences in the accessibility of health services, depending on the health condition, were found, with majority of women receiving basic antenatal services, whilst people suffering from diabetes or hypertension received no medication and did not know how to access care. Consequently, the burden of NCDs remains hidden to a large degree. These findings identify gaps in current access to healthcare but also highlight some potential opportunities for delivery of affordable community-based health services.

The choice of healthcare provider was found to be strongly fragmented, with different types of providers being accessed depending on the health condition and frequent changes of providers between multiple consultations. The public sector as a sole source of care was only preferred within maternal health (57% of women); otherwise the majority of care (62%–90% of consultations across conditions) was obtained from private or a mixture of public and private providers. Our findings are consistent with other studies that found that private providers, whilst significantly more expensive, were favoured as they were perceived to provide a better and a safer service ^22, 27, 33^. Moreover, previous studies have also commented on poor user experience within the public health system, including time-inefficiency and ill-treatment by clinical staff, reinforcing preferences for private care ^34^. Insight into how slum populations navigate the heterogeneous environment of healthcare providers, in the context of differing health conditions and needs, is crucial for effective healthcare planning to ensure universal access to high quality healthcare for these vulnerable populations.

Our finding that the choice of healthcare provider is associated with a number of socio-demographic and lifestyle factors is echoed in a number of other studies ^35– 37^. Surprisingly, the level of education was not found to be associated with provider choice; this might be attributed to the higher average education level in the community, compared to other studies ^33, 38^. Healthcare expenditure on individual consultations was found to be vaguely associated with socio-demographics – household income for men and duration of residency in the community for women. Women who are recent migrants in the community have limited knowledge of health providers and weak social networks, which has been highlighted as a barrier to health access ^27^. The fact that maternal health expenditure showed no association with economic factors could be explained by the complex combination of cultural and social circumstances that have been reported to influence maternal health decision making, e.g. the number of children, tradition, the influence of mothers-in-law ^39– 42^. There are a number of possible limitations to this study which should be considered. The study was cross-sectional in design, making the evaluation of temporal relationships between socio-demographic factors and healthcare decisions impossible. Additionally, socio-demographic and lifestyle characteristics, as well as disease prevalence, are self-reported by participants, which might have introduced information and/or recall bias as well as substantial amount of under-reporting of disease incidence, particularly NCDs, as most people reported to never have had their blood sugar or blood pressure measured. The sample size was found to be insufficient to investigate other factors likely to be affecting healthcare choices, such as the geographical distribution of participant’s households and proximity to public/private facilities. More detailed surveying of provider availability and quality of services for each area is required to further map any spending patterns in relation to service supply. Finally, given the choice of study population and socio-economic specifics of Mumbai (a more economically developed city), findings are not directly generalisable across slum communities.

Slum communities, who navigate a complex landscape of over-burdened public and unregulated private providers, are often victims of higher mortality and morbidity, as well as crippling out-of-pocket expenditure that sends them deeper into poverty ^17, 33^. This study provides a rare insight into the factors associated with healthcare choices amongst slum communities in Mumbai. Findings highlight stark differences between services for well-known health conditions (e.g. maternal health) and less known, emerging problems (diabetes and hypertension). This suggests that, to achieve universal health coverage, integration of new services into existing care delivery channels might be advantageous. Although people in these communities are poor, their health expenditure was found not to be associated with income and spending on private providers was very common, especially on providers operating in the community. This is consistent with other studies where private services were favoured as they did not result in a daily wage loss ^43^. This suggests that public-private partnerships that strengthen capacity for community-based care may be an appropriate model that allows for better regulation of providers, whilst satisfying convenience requirements, as discussed in the literature ^44– 46^. The implementation of such programmes should be conducted in the context of the National Urban Health Mission of the Government of India, providing a framework for the identification of both listed and unlisted slums ^47, 48^. In order to make progress towards universal health coverage to equitable and quality health services, strategic planning and innovation within the Indian healthcare system will be crucial and slum populations must not be left behind. Hence, further research focused on healthcare within urban slum communities is greatly needed. 

## Data availability

Data collected in this study are available on OSF: http://doi.org/10.17605/OSF.IO/WPD4V ^49^

No identifying information was collected as part of this study and hence the dataset is fully anonymised.

Data are available under the terms of the Creative Commons Zero “No rights reserved” data waiver (CC0 1.0 Public domain dedication).
