# Supplementary material for: Healthcare choices in Mumbai slums: A cross-sectional study
Source: Wellcome Open Res. 2018 Jul 6;2:115. Originally published 2017 Dec 5. [Version 2] doi: 10.12688/wellcomeopenres.13127.2 (PMC6039940; doi:10.12688/wellcomeopenres.13127.2)
Supplement: Peer review history

# Peer review: Referee response for version 2

**DOI:** 10.21956/wellcomeopenres.16002.r33450

**Published:** 2018-07-30

**Authors:** Siddharth Agarwal, Kanupriya Kothiwal

**Affiliations:** 1 Urban Health Resource Centre (UHRC), Indore, India

Thank you for responding to two of the three main suggestions made in the peer review, which have strengthened the manuscript. It is heartening to see these improvements.

 The authors are urged to not omit a brief description of the Urban Healthcare system in Mumbai, since this will enhance the value of the paper immensely. Reducing words in the discussion section and any other section of the manuscript the authors deem appropriate will enable inserting a succinct description of the public sector urban healthcare delivery system of the slums in Mumbai (offered by Municipal corporation of Greater Mumbai and the State Government of Maharashtra) that they studied in Bandra, Khar, Santa Cruz and Andheri. Stating that this has already been described reflects relinquishing the authors’ responsibility to previously published work of other researchers.

 With a crisp, short description of the urban healthcare system in Mumbai, the paper will be very good for the readers of Wellcome Open Research.

We have read this submission. We believe that we have an appropriate level of expertise to confirm that it is of an acceptable scientific standard, however we have significant reservations, as outlined above.

# Peer review: Referee response for version 2

**DOI:** 10.21956/wellcomeopenres.16002.r33452

**Published:** 2018-07-16

**Authors:** Gagandeep Kang

**Affiliations:** 1 Division of Gastrointestinal Sciences, Christian Medical College, Vellore, India

I have read this submission. I believe that I have an appropriate level of expertise to confirm that it is of an acceptable scientific standard.

# Peer review: Referee response for version 2

**DOI:** 10.21956/wellcomeopenres.16002.r33451

**Published:** 2018-07-09

**Authors:** Luc P. de Witte

**Affiliations:** 1 School for Health and Related Research, Centre for Assistive Technology and Connected Healthcare (CATCH), University of Sheffield, Sheffield, UK

I am very happy to see that the authors have made some relevant changes to their paper, and that they carefully discuss the issues raised in my earlier review in the present version. The article presents important and interesting data about the health situation and healthcare in urban slums and has the potential to support healthcare organisations in developing optimal strategies to provide essential healthcare in these settings.

I have read this submission. I believe that I have an appropriate level of expertise to confirm that it is of an acceptable scientific standard.

# Peer review: Referee response for version 1

**DOI:** 10.21956/wellcomeopenres.14239.r30077

**Published:** 2018-02-27

**Authors:** Siddharth Agarwal, Kanupriya Kothiwal

**Affiliations:** 1 Urban Health Resource Centre (UHRC), Indore, India

Overall, this is a well written paper that has studied healthcare among an important and rapidly growing segment of the World’s and India’s population.

 Below are two strengths of and three major suggestions for this paper and then follow more detailed section-wise review comments and suggestions.

 Strengths:

1. The methods and results are clearly described.
2. The authors have highlighted the reported level of access to healthcare services of slum women and men. The paper also insightfully describes the different types of healthcare providers accessed by slum women and men for different health conditions.

 

 Suggestions:

1. This paper will vastly benefit by mentioning Government of India’s mandate and suggested methods of identifying all slums (listed as well as unlisted) and slum enumeration methods utilised in Indian cities.
2. The authors should describe the existing public sector healthcare system of Municipal Corporation of Greater Mumbai and State Government of Maharashtra in the four areas of Mumbai where the sample was drawn.
3. Strengthening and broadening the discussion about factors associated with slum women and men accessing private healthcare providers will enhance the significance of this paper by referring to slum healthcare literature from Indian Sub-continet.

 

 More detailed section wise review:

**Introduction**:

1. The authors are encouraged to briefly describe how this paper contributes information that is not already available to the larger body of knowledge; and how the data from this research stored in digital format in Centre for the Study of Social Change (CSSC) is contributing to the community that was studied and where CSSC works towards grassroots social development focused on strengthening primary healthcare services.
2. Identifying slums as spatial entities is a vital concept for understanding and strengthening healthcare in slums. This aspect should be strengthened in the paper by mentioning Government of India’s mandate and suggested methods of identifying all slums (listed as well as unlisted) and slum enumeration methods utilised in Indian cities. The Draft Final Report of the Urban Health Task Force of the Ministry of Health and Family Welfare, Government of India recommended “mapping of both listed and unlisted slums” ^1^. The National Urban Health Mission of the Government of India, which is based on the recommendations of the aforementioned task force, mandates inclusion of “urban poor population living in listed and unlisted slums; all other vulnerable population such as homeless, rag-pickers, street children, rickshaw pullers, construction and brick and lime kiln workers, sex workers, and other temporary migrants” (page 5) ^2^ and “mapping of listed and unlisted slums” (page 76) ^2^. There is evidence from literature that community enumeration and assessment from Indian cities showed a substantial proportion of unlisted slums (Vlahov et al, 2011 ^3^). The National Sample Survey (page (i) of the report) found that 59.9% slums were “non-notified” ^4^.
3. The scarcity of data about healthcare access of slum populations is crucial to address. At the same time, the authors could mention available health access data about urban disadvantaged populations from literature such as from “Health and Living Conditions in Indian Cities: National Family Health Survey (NFHS-3) India 2005-06” ^5^ and “The State of Urban Health in India: Comparing the poorest quartile to the rest of the urban population in selected states and cities (2011)” ^6^.
4. The authors should succinctly describe the public sector urban healthcare delivery system of the slums in Mumbai (offered by Municipal corporation of Greater Mumbai and the State Government of Maharashtra) that they studied in Bandra, Khar, Santa Cruz and Andheri, to enable the readers of Wellcome Open Research better understand the findings and discussion related to choice of healthcare providers well described in this paper.

**Methods and Results**

1. Study methods and results are well described are clear.
2. The authors have highlighted the reported level of access to healthcare services of slum women and men. The paper also insightfully describes the different types of healthcare providers accessed by slum women and men for different health conditions.
3. If the authors undertake any future studies on similar themes in any country, they would do well to complement quantitative data along with qualitative findings.

**Discussion**

1. The discussion section captures the findings very well.
2. The authors should consider describing before “this suggests that public private partnerships…” in paragraph 5 of the discussion, additional factors that affect health provider choice of slum dwelling women and men utilising slum healthcare literature from South Asia. This strengthening and broadening could include a) availability of private providers in or near community in the evening (Adams et al. 2015 ^7^); b) long distance (also mentioned in this paper), time consuming procedure and rude behaviour during seeking healthcare from Government facilities (Baruah and Pandav, 2011 ^8^); c) low income, compulsion to work for survival (Agarwal et al. 2009 ^9^), wage loss in seeking public sector healthcare (Saw, et al).

**A suggestion:** The authors are encouraged to consider revising the language of sentences such as “Participants were allowed to select multiple providers per health condition.” It would be worthwhile to describing what must have actually been done by the data collectors, who have been working long term in the community.

We have read this submission. We believe that we have an appropriate level of expertise to confirm that it is of an acceptable scientific standard, however we have significant reservations, as outlined above.

## References

[1] Draft Final Report of the Task Force to Advise the National Rural Health Mission on “Strategies for Urban Health Care”. *Ministry of Health and Family Welfare Government of India, New Delhi*.2006;25

[2] National Urban Health Mission Framework for Implementation. *Ministry of Health and Family Welfare Government of India*.2013;

[3] VlahovDAgarwalSRBuckleyRMCaiaffaWTCorvalanCFEzehACFinkelsteinRFrielSHarphamTHossainMde Faria LeaoBMboupGMontgomeryMRNetherlandJCOmpadDCPrasadAQuinnATRothmanASatterthwaiteDEStansfieldSWatsonVJ: Roundtable on Urban Living Environment Research (RULER). *J Urban Health*.2011;88(5) : 10.1007/s11524-011-9613-2 793-857 10.1007/s11524-011-9613-2 21910089PMC3191208

[4] National Sample Survey Report, 69th Round. *Ministry of Statistics and Programme Implementation, Government of India*.2012;

[5] GuptaKArnoldFLhungdimH: Health and Living Conditions in Eight Indian Cities. National Family Health Survey (NFHS-3) India 2005-2006. *Mumbai: International Institute for Population Sciences*.2009;

[6] AgarwalS: The state of urban health in India; comparing the poorest quartile to the rest of the urban population in selected states and cities. *Environment and Urbanization*.2011;23(1) : 10.1177/0956247811398589 13-28 10.1177/0956247811398589

[7] AdamsAMIslamRAhmedT: Who serves the urban poor? A geospatial and descriptive analysis of health services in slum settlements in Dhaka, Bangladesh. *Health Policy Plan*.2015;30 Suppl 1: 10.1093/heapol/czu094 i32-45 10.1093/heapol/czu094 25759453PMC4353891

[8] BaruaNPandavCS: The allure of the private practitioner: is this the only alternative for the urban poor in India?. *Indian J Public Health*.55(2) : 10.4103/0019-557X.85242 107-14 10.4103/0019-557X.85242 21941045

[9] AgarwalSSrivastavaAKumarS: Urban health in developing countries. *Perspectives of Knowledge Management in Urban Health*.2010;61-94

[10] Skordis-WorrallJPaceNBapatUDasSMoreNSJoshiWPulkki-BrannstromAMOsrinD: Maternal and neonatal health expenditure in Mumbai slums (India): a cross sectional study. *BMC Public Health*.2011;11: 10.1186/1471-2458-11-150 150 10.1186/1471-2458-11-150 21385404PMC3061914

# Response

**Published:** 2018-07-04

**Authors:** Elina Naydenova

**Affiliations:** University of Oxford, UK

Thank you very much for your insightful comments and feedback. We have addressed your feedback one by one below.

**Introduction**

 1. We have amended the introduction to highlight how our research supplements existing knowledge.

*This study examined the disease burden, health awareness and services, and healthcare choices reported by residents across 13 slums in Mumbai, India.* *To provide insights into the heterogeneous nature of healthcare choices, the study focused on parents of young children who are likely to have recently encountered a number of health challenges including maternal health, common health conditions, and early-onset NCDs. These insights are of high relevance to integrated health programmes looking to align incentives behind traditionally verticalized services.*

 2. Thank you very much for highlighting the importance of identifying slums as spatial entities and the important work conducted by the Government of India in this context. We have made changes to emphasise this in the discussion of the paper.

*This suggests that public-private partnerships that strengthen capacity for community-based care may be an appropriate model that allows for better regulation of providers, whilst satisfying convenience requirements, as discussed in the literature ^– 43^. The implementation of such programmes should be conducted in the context of the National Urban Health Mission of the Government of India, providing a framework for the identification of both listed and unlisted slums ^49,50^.*

 3. References to existing sources of health access data in slum populations have been added to the introduction.

*Agarwal has published a comprehensive overview of the healthcare landscape in Indian slums and the National Family Health Survey, conducted by the government of India, has reported on incidence statistics, particularly in the context of maternal and child health ^45, 46^. Beyond these, health statistics and access to care data about slum populations are sparse, with the vast inter- and intra- slum variability rarely captured in formal reports ^15^^– 18^.*

 4. Thank for highlighting the importance of the readers’ understanding of the health system context, we completely agree with your point. Whilst the word limit of this publication does not allow us to cover this in great lengths, we have highlighted references to a few other studies conducted by the CSSC team with the same population that describe the context in more detail.

*The study population and health system context has previously been described, ^27^*

**Methods and results**

 1. “ *Study methods and results are well described are clear.*”

 Thank you very much for acknowledging this.

 2. “ *The authors have highlighted the reported level of access to healthcare services of slum women and men. The paper also insightfully describes the different types of healthcare providers accessed by slum women and men for different health conditions.*”

 Thank you very much for your complimentary feedback.

 3. “ *If the authors undertake any future studies on similar themes in any country, they would do well to complement quantitative data along with qualitative findings.*”

 We completely agree with your suggestion regarding complimenting quantitative findings with qualitative ones and are committed to doing this in future studies. We hope that other research teams follow your recommendation too.

**Discussion**

 1. “ *The discussion section captures the findings very well.*”

 Thank you very much.

 2. We completely agree with your suggestion and have amended the discussion to highlight other factors influencing healthcare choices as per the existing literature.

*Other studies have suggested that slum communities access care from a mixture of public and private providers, with distance, household decision-making structure and perceived quality of care being important factors in their decision ^20^^– 23^.*

 ….

*Our findings are consistent with other studies that found that private providers, whilst significantly more expensive, were favoured as they were perceived to provide a better and a safer service ^21^^, 26, 32^. Moreover, previous studies have also commented on poor user experience within the public health system, including time-inefficiency and ill-treatment by clinical staff, reinforcing preferences for private care ^47^.*

 ….

*Although people in these communities are poor, their health expenditure was found not to be associated with income and spending on private providers was very common, especially on providers operating in the community. This is consistent with other studies where private services were favoured as they were less likely to result in a daily wage loss ^48^.*

 Kind regards,

 Elina Naydenova

# Peer review: Referee response for version 1

**DOI:** 10.21956/wellcomeopenres.14239.r30080

**Published:** 2018-02-15

**Authors:** Gagandeep Kang

**Affiliations:** 1 Division of Gastrointestinal Sciences, Christian Medical College, Vellore, India

This article reports results of a reasonably designed healthcare utilization survey in Mumbai slums. It would have been helpful if the investigators had designed the study to assess the services which the government has focused on delivering (maternal and child health including immunization and nutrition services) and those that have been emphasized as facility based care (emergencies, injuries, need for in-patient care). This would be helpful in evaluating whether the governmental goals are being met, even in part (e.g. antenatal care, facility delivery and immunization, nutrition) and where the private sector is filling a gap or replacing government services.

 The lack of use of pharmacies and alternative medicine is a surprising finding and may be reflective of either Mumbai or the youth and education of participants which are different from other healthcare utilization surveys in India. This is likely because of the need to enroll those who have recently had or will shortly have a birth in the family.

I have read this submission. I believe that I have an appropriate level of expertise to confirm that it is of an acceptable scientific standard, however I have significant reservations, as outlined above.

# Response

**Published:** 2018-07-04

**Authors:** Elina Naydenova

**Affiliations:** University of Oxford, UK

Thank you very much for your excellent suggestion regarding the evaluation of government services in the urban slum communities. We will most definitely incorporate this in future research and encourage colleagues to do so too. Such insights would be complimentary to the work conducted here which focused on factors influencing healthcare choices among urban slum dwellers. As you highlight in your comments, healthcare delivery in such communities is fragmented across public and private providers and previous research has sought to evaluate gaps in public health delivery filled in by private services [1,2,3]. Our research aimed to capture unique insights regarding the complex drivers behind residents’ health choices, investigated in the context of a range of socio-economic factors.

 Your comment about generalisability is very insightful and we have added comments in the discussion section to reflect this (see underlined text below). We completely agree that findings are not necessarily generalisable across India and are driven by a number of socio-demographic factors associated with the urban slum population of Mumbai as well as the inclusion criteria of the study.

***Discussion:***

*Our study presents findings on healthcare challenges and choices in a number of slum communities across Mumbai. The study focused on parents of young children as a subsection of the population experiencing a range of health challenges, where healthcare choices are influenced by different socio-economic factors. A number of important differences with previous studies, focused on other demographic groups, were found, including differences in education levels and health awareness, as discussed below. This demographic is highly relevant for organisations designing integrated health programmes (e.g. NCD-MCH programmes) as well as forward-looking health policy development that might target this demographic as a household entry point for health interventions (more than 65% of participants reported to be responsible for financial decisions including healthcare of the household).*

**

*…*

*Additionally, socio-demographic and lifestyle characteristics, as well as disease prevalence, are self-reported by participants, which might have introduced information and/or recall bias as well as substantial amount of under-reporting of disease incidence, particularly NCDs, as most people reported to never have had their blood sugar or blood pressure measured.* *The sample size was found to be insufficient to investigate other factors likely to be affecting healthcare choices, such as the geographical distribution of participant’s households and proximity to public/private facilities. More detailed surveying of provider availability and quality of services for each area is required to further map any spending patterns in relation to service supply. Finally, given the choice of study population and socio-economic specifics of Mumbai (a more economically developed city), findings are not directly generalisable across slum communities.*

  [1] Banerjee A, Bhawalkar JS, Jadhav SL, Rathod H, Khedkar DT. Access to Health Services Among Slum Dwellers in an Industrial Township and Surrounding Rural Areas: A Rapid Epidemiological Assessment. *Journal of Family Medicine and Primary Care*. 2012;1(1):20-26. doi:10.4103/2249-4863.94444.

 [2] Skordis-Worrall J, Pace N, Bapat U, Das S, More NS, Joshi W, Pulkki-Brannstrom AM, Osrin D. Maternal and neonatal health expenditure in Mumbai slums (India): A cross sectional study. *BMC Public Health* 2011; 11:150. https://doi.org/10.1186/1471-2458-11-150

 [3] Gupta I, Guin, P. Health Status and Access to Health Services in Indian Slums. *Health. *2015; 7, 245-255. doi: 10.4236/health.2015.72029.

 Kind regards,

 Elina Naydenova

# Peer review: Referee response for version 1

**DOI:** 10.21956/wellcomeopenres.14239.r28674

**Published:** 2017-12-27

**Authors:** Luc P. de Witte

**Affiliations:** 1 School for Health and Related Research, Centre for Assistive Technology and Connected Healthcare (CATCH), University of Sheffield, Sheffield, UK

Research into the health issues in urban slums is important. As the authors state the literature on this topic is very scarce, while the number of people living in slums is increasing. From this point of view the paper is an important contribution. I do, however, have some concerns about the methodology applied that should be taken into account when interpreting the results.

 The most important concern is that the study population clearly is a selection of relatively young people with surprisingly high levels of education. By including only people who have had children within the last 5 years or were expecting a child the authors excluded a large part of the slum population. For studying maternal health issues this selection makes perfect sense, but the aim of the study was also to examine the disease burden in general, with maternal healthcare being one of the fields of interest. This chosen selection has a strong impact on the results regarding NCDs, almost certainly leading to a underestimation of the prevalence of diseases like hypertension and diabetes.  

 Another concern regards the fact that, understandably, only self-reported prevalence data were collected. This also leads to an underestimation of prevalence data, especially for hypertension and diabetes, simply because these diseases often go without clear complaints. The fact that only less than half of the people report the have ever measured their blood pressure and only less than a quarter their blood sugar suggests that the prevalence figures presented may easily be double in reality. 

 I would expect the above concerns to be discussed in the discussion section of the paper. 

 I agree with the authors that much more research is needed into the health profiles of people living in slums. For proper planning and coordination of health services to cope with the health issues in slums, we do need more precise and 'objective' data then those presented in this paper. But again, keeping the concerns mentioned in mind, this paper is an important contribution to this field. 

I have read this submission. I believe that I have an appropriate level of expertise to confirm that it is of an acceptable scientific standard, however I have significant reservations, as outlined above.

# Response

**Published:** 2018-07-04

**Authors:** Elina Naydenova

**Affiliations:** University of Oxford, UK

Thank you for your great comments and feedback. Your comments about the demographic profile of the study population and the self-reported nature of observations raise very important point. We have made changes to the introduction and discussion sections to clarify and highlight that:

1. This study focuses on a population of parents of young children as this demographic is likely to face a wide range of health challenges, from common health problems, through maternal care, to early onset of NCDs. This group is particularly important for organisations designing integrated health services (e.g. MCH-NCD). As you very rightly emphasise, this demographic is not representative for the whole slum population when it comes to incidence rates of NCDs. For that, a much larger population sample, capturing people of older age too, will be required.
2. Findings are therefore not directly generalisable across slum populations.
3. Disease incidence findings in the study population should mostly be interpreted as an indication of the hidden burden of diseases such as NCDs in slums populations, as most people reported to have not had screening tests such as blood sugar and blood pressure.
4. The self-reported nature of observations is likely to introduce bias and a degree of under-reporting when it comes to disease incidence.

Changes to the publication are underlined below.

***Introduction:***

*This study examined the disease burden, health awareness and services, and healthcare choices reported by residents across 13 slums in Mumbai, India. To provide insights into the heterogeneous nature of healthcare choices, the study focused on parents of young children who are likely to have recently encountered a number of health challenges including maternal healthcare, common health conditions, and early-onset NCDs. These insights are of high relevance to integrated health programmes looking to align incentives behind traditionally verticalized services.*

***Methods:***

*The study focused on parents of young children in order to provide direct comparison between people’s choices regarding a range of healthcare challenges. The demographic captured in this study is indicative of future challenges facing urban slum populations as their current choices and risk factors will directly affect their and their children’s health. Moreover, integrated health solutions targeting early risk factors of NCDs, as highlighted in the Introduction, would target this demographic group.*

***Discussion:***

*Our study presents findings on healthcare challenges and choices in a number of slum communities across Mumbai. The study focused on parents of young children as a subsection of the population experiencing a range of health challenges, where healthcare choices are influenced by different socio-economic factors. A number of important differences with previous studies, focused on other demographic groups, were found, including differences in education levels and health awareness, as discussed below. This demographic is highly relevant for organisations designing integrated health programmes (e.g. NCD-MCH programmes) as well as forward-looking health policy development that might target this demographic as a household entry point for health interventions (more than 65% of participants reported to be responsible for financial decisions including healthcare of the household).*

*…*

*Differences in the accessibility of health services, depending on the health condition, were found, with majority of women receiving basic antenatal services, whilst people suffering from diabetes or hypertension received no medication and did not know how to access care. Consequently, the true burden of NCDs remains hidden to a large degree.*

*…*

*Additionally, socio-demographic and lifestyle characteristics, as well as disease prevalence, are self-reported by participants, which might have introduced information and/or recall bias as well as substantial amount of under-reporting of disease incidence, particularly NCDs, as most people reported to never have had their blood sugar or blood pressure measured.* *The sample size was found to be insufficient to investigate other factors likely to be affecting healthcare choices, such as the geographical distribution of participant’s households and proximity to public/private facilities. More detailed surveying of provider availability and quality of services for each area is required to further map any spending patterns in relation to service supply. Finally, given the choice of study population and socio-economic specifics of Mumbai (a more economically developed city), findings are not directly generalisable across slum communities.*

 Kind regards,

 Elina Naydenova
